# A Comparative Analysis of the Ocular Microbiome: Insights into Healthy Eyes and Anophthalmic Sockets

**DOI:** 10.3390/microorganisms12112298

**Published:** 2024-11-12

**Authors:** Francisco Zamorano-Martín, Guillermo Chumaceiro, Pablo Navarro-Torres, Davide Borroni, Facundo Urbinati, Ángel Molina, Andreu Paytuví-Gallart, Carlos Rocha-de-Lossada

**Affiliations:** 1 Departament of Ophthalmology, Hospital Universitario Virgen de las Nieves, 18016 Granada, Spain; zamoranomartinfrancisco@gmail.com (F.Z.-M.);; 2 Department of Radiology and Physical Medicine, Ophthalmology and Otorhinolaryngology, Ophthalmology Area, Faculty of Medicine, University of Malaga, 29016 Malaga, Spain; 3 Granada Vision and Eye Research Team (VER), Instituto de Investigación Biosanitaria ibs.GRANADA, 18016 Granada, Spain; 4 Sequentia Biotech SL, Carrer del Dr. Trueta, 179, 08005 Barcelona, Spain; gchumaceiro@sequentiabiotech.com (G.C.); amolina@sequentiabiotech.com (Á.M.); apaytuvi@sequentiabiotech.com (A.P.-G.); 5 Department of Ophthalmology, Riga Stradins University, LV-1007 Riga, Latvia; 6 Departament of Ophthalmology, Hospital Universitario Torrecárdenas, 04001 Almeria, Spain; facundou10@gmail.com; 7 Qvision, Ophthalmology Department, VITHAS Almeria Hospital, 04009 Almeria, Spain; carlosrochadelossada5@gmail.com; 8 Ophthalmology Department, VITHAS Malaga, 29016 Malaga, Spain; 9 Departament of Ophthalmology, Hospital Regional Universitario de Malaga, 29010 Malaga, Spain; 10 Departamento de Cirugía, Universidad de Sevilla, Área de Oftalmología, 41001 Sevilla, Spain

**Keywords:** anophthalmic socket, microbiota, microbiome, proteobacteria, firmicutes, ocular surface, eye

## Abstract

The purpose of this study is to characterize the ocular surface microbiota of patients with an anophthalmic cavity. An eNAT with 1 mL of Liquid Amies Medium was used to collect samples. Microbial DNA from anophthalmic socket and healthy fellow control eye samples was isolated and sequenced. Raw reads were analyzed with GAIA (v 2.02). The richness and Shannon alpha diversity metrics, as well as Bray–Curtis beta diversity and Wilcoxon signed-rank test values, were computed with R packages such as phyloseq, mia, or DESeq2 to allow for microbiome analysis. Principal coordinate analysis (PCoA) was performed using the function plotReducedDim from the R package scater. The different taxonomic profiles were described under the concept of eye community state type (ECST). The microbiomes of both eyes from 25 patients with an anophthalmic cavity were analyzed in this study. While the microbial communities of paired eyes from the same patients showed notable dissimilarity, no consistent patterns emerged when comparing healthy eyes to anophthalmic sockets. Alpha diversity values did not significantly differ between healthy eyes and anophthalmic socket samples, though there was considerable variability within each group. Notably, anophthalmic socket samples generally exhibited lower abundances of genera such as *Staphylococcus*, *Enterococcus*, *Paenibacillus*, and *Sediminibacterium* compared to their healthy counterparts. Microbial variability between healthy eyes and anophthalmic sockets may be due to anatomical differences. Further research is needed to determine whether patients without anophthalmic sockets exhibit similar microbiome patterns in both eyes.

## 1. Introduction

The loss of an eye, resulting in the creation of an anophthalmic socket, leads to significant changes in a patient’s daily life, including the need for the consistent maintenance of both the ocular prosthesis and the anophthalmic cavity. Understanding these changes is crucial to improving patient care. A key area of growing interest is the study of the ocular surface microbiome (OSM), which plays a vital role in maintaining the health of the ocular surface and protecting it from pathogens. Changes in the OSM may contribute to conditions such as discomfort, dryness, and inflammation, common in patients with anophthalmic sockets [1].

The human body is colonized by a vast array of microorganisms—bacteria, archaea, fungi, and viruses—that together form a complex community known as the human microbiome. Advances in diagnostic techniques, such as metagenomics, have enabled a deeper understanding of the microbial populations in the human body, including the ocular surface [2]. Unlike traditional culture-based methods, which are limited in scope, metagenomic sequencing allows for the rapid and comprehensive identification of microbial species, providing insights into both commensal and pathogenic microorganisms [3]. Previous research has highlighted how alterations in the OSM can be linked to various ocular conditions. For example, studies have shown that keratoconus, contact lens wear, and dry eye disease are associated with significant shifts in microbial composition. However, it remains unclear whether these microbiota changes are a cause or consequence of such conditions. Exploring the OSM in anophthalmic patients offers an opportunity to further investigate these dynamics and understand the potential role of dysbiosis in the development of dry anophthalmic socket syndrome (DASS) [4,5,6,7,8,9,10,11]. The objective of this study is to characterize the OSM in patients with anophthalmic sockets using 16S rRNA metagenomic sequencing and compare it with the microbiota of a contralateral healthy eye. By identifying specific microbial changes in these patients, we aim to contribute to the growing body of knowledge on how microbiota alterations may influence ocular surface health.

## 2. Materials and Methods

This study was approved by the institutional review board of the Hospital Universitario Virgen de las Nieves (Granada, Spain) with the code Metagenomic 1844-N-22. Patients were recruited in the Ophthalmology department of Hospital Regional Universitario de Málaga from January to October 2022.

This descriptive cross-sectional study followed the tenets of the Declaration of Helsinki, and informed consent was obtained from human subjects involved in this study. Inclusion criteria were (1) patients with an anophthalmic socket and eye prosthesis for at least 6 months and (2) age ≥ 18 years. Exclusion criteria included (1) patients who used anti-inflammatory or antibiotic medication in either eye during the last week; (2) patients with complications of the anophthalmic socket that could be related to discomfort symptoms such as conjunctival cyst, implant exposure, or chronic conjunctivitis; (3) patients with an active infection in either eye; (4) patients with eyelid malposition; and (5) patients wearing bilateral, defective, or poor-fitting prosthetic eyes.

### 2.1. Sampling Technique, DNA Extraction, PCR Amplification, Library Preparation, and Amplicon Sequencing

Samples were collected with an eNAT with 1 mL of Liquid Amies Medium (Copan, Brescia, Italy). The eNAT swab was first applied on the inferior surface of the eye surface and moved two times, “limbus to fornix to limbus” in the healthy eye and with a wide movement through the cavity and the fornix in the anophthalmic socket [12]. Then, the swab was placed in the tube with Liquid Amies Medium. Neither fluorescein nor anesthetic drops were used to avoid influences on the eye microbiota. Microbial DNA from anophthalmic socket and healthy control samples was standardly isolated using the QIAamp DNA Microbiome Kit (Qiagen, Hilden, Germany) following the manufacturer’s instructions. The microbial DNA was checked and quantified using Genomic DNA ScreenTape (Agilent Technologies, Santa Clara, CA, USA) at Agilent TapeStation 4150. DNA libraries were prepared using the Ion 16S Metagenomics Kit (Thermo Fisher, Waltham, MA, USA), and sequencing was performed on an Ion Torrent S5 system, following standard protocols. The primers used were part of the Ion 16S Metagenomics Kit. An input DNA amount of 0.5 ng was used for library preparation. Amplification was completed using two primer sets to amplify the hypervariable regions of the 16S rDNA gene. Amplified products were purified using AMPure XP beads (Beckman Coulter Agencourt, Thermo Fisher, Waltham, MA, USA) and end-repaired for barcode ligation. Later, libraries were verified for quality and quantified using Agilent High Sensitivity D1000 ScreenTape (Agilent Technologies, USA). Equimolar libraries were pooled together at the final concentration of 5 pM. Template preparations were performed with Ion Chef according to the Ion 540 Kit-Chef protocol (Thermo Fisher, Waltham, MA, USA). The amplicon libraries were sequenced on a 540-chip using the Ion Torrent S5 system (Thermo Fisher, Waltham, MA, USA) according to the supplier’s instructions. After sequencing, low-quality and polyclonal sequences were filtered out by the Ion software 5.14, and the gained data were submitted to the dedicated software for analysis. Nuclease-free water and all the reagents used in this experiment were processed as a negative control. Contamination with extraneous bacterial DNA in DNA extraction kit reagents and the wider ambulatory and laboratory environment was minimized by collecting control and experimental samples at the same time, under the same conditions, and handling them together.

### 2.2. Data Analysis

Raw reads were analyzed with the metagenomics software GAIA (ver. 2.0.1) (https://metagenomics.sequentiabiotech.com) to taxonomic tables at different levels. The present study focused on genera. To filter putative false positives, only those genera supported by at least two reads in at least two samples were considered for the downstream analyses.

The richness and Shannon alpha diversity metrics, along with Bray–Curtis beta diversity and Wilcoxon signed-rank test values, were computed using R packages such as “mia” (ver. 1.12.0) and “DESeq2” (ver. 1.44.0) to facilitate microbiome analysis. Bray–Curtis beta diversity measures the dissimilarity between two samples based on the abundance of different microbial genera. It provides a value representing how distinct the microbial communities are, which is then visualized through PCoA. Differential abundance analysis (DAA) was performed using “DESeq2” to identify genera that were significantly more or less abundant between groups. To account for intra-individual variability, a paired design was implemented by including patient identifiers in the DESeq2 model. This approach allowed us to accurately pinpoint specific microbial changes associated with different conditions. Principal coordinate analysis (PCoA) was conducted using the “plotReducedDim” function from the R package “scater” (ver. 1.32.0) to visualize patterns in beta diversity among the samples. PCoA is a technique used to visualize differences in microbial communities. It reduces complex data into principal axes, allowing us to plot and compare how similar or different the microbial profiles are between samples.

## 3. Results

### 3.1. Taxonomic Profiling

**Figure 1:**
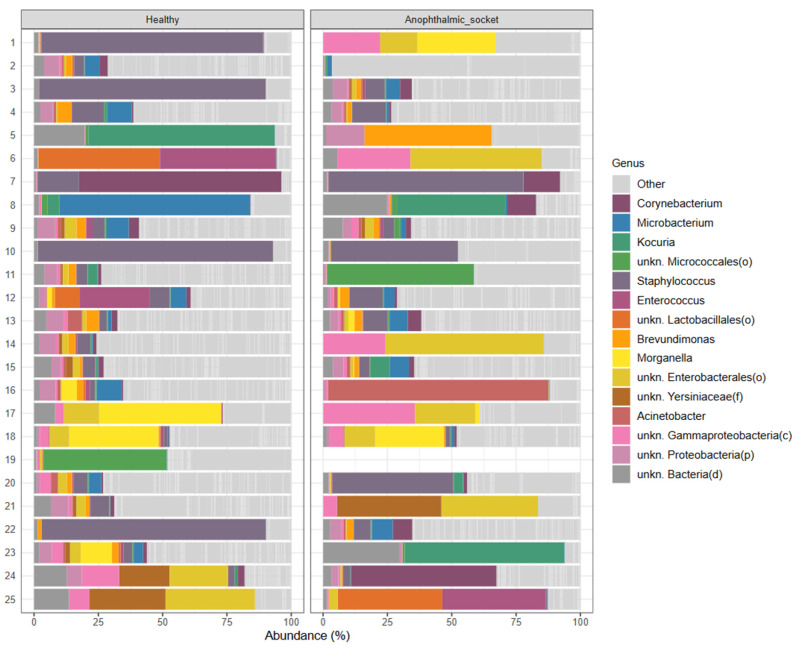
A taxonomy barplot of the microbiota of healthy eyes (**left**) and the corresponding contralateral eye with an anophthalmic socket (**right**). The barplot represents the relative abundance of the top 15 genera across samples and the group “Other” summing all genera with an abundance below 1%. Data from the anophthalmic socket from patient 19 were missing.

To accurately depict microbiome composition for each sample, we generated a barplot representing the relative abundance of identified genera (Figure 1). For clarity, the plot displays only the 15 most abundant genera across all samples, with those comprising less than 1% grouped under “Other”.

On average, *Staphylococcus*, *Corynebacterium*, *Brevundimonas*, and *Microbacterium* are the most abundant genera across all samples, with *Staphylococcus* being particularly prominent in healthy eyes and there being a broader mix of these genera in the anophthalmic socket eyes.

As the barplot shows, there is substantial variability in microbial composition between healthy eyes and anophthalmic sockets, with notable differences among the top 15 genera. Importantly, the most abundant genera in healthy eyes are not consistently mirrored in their paired anophthalmic sockets, underscoring significant variability and a lack of similarity between eyes from the same patient.

We also predicted the eye community state type (ECST) for both eyes (healthy and contralateral anophthalmic sockets) from the same individual based on the ECST profiles previously described by Borroni et al. [5]. One healthy eye was classified as ECST 1, with the contralateral anophthalmic socket classified as ECST 2, while another healthy eye was classified as ECST 1, with the socket classified as ECST 9. Two healthy eyes were classified as ECST 2, with their contralateral sockets classified as ECST 1. No samples were classified as ECST 3, ECST 4, or ECST 7. One healthy eye was classified as ECST 5, with the socket classified as ECST 2, while another pair of healthy and socket eyes were both classified as ECST 5. Two healthy eyes classified as ECST 5 had contralateral sockets classified as ECST 6. Two healthy eyes classified as ECST 6 had contralateral sockets classified as ECST 1, while another pair had a healthy eye classified as ECST 6 and the socket classified as ECST 5. Eight healthy eyes and their contralateral sockets were both classified as ECST 6, and two healthy eyes classified as ECST 6 had their contralateral sockets classified as ECST 9. One healthy eye classified as ECST 8 had a contralateral socket classified as ECST 9. Finally, one healthy eye classified as ECST 9 had its contralateral socket classified as ECST 5 and another as ECST 6.

### 3.2. Alpha Diversity Analysis

We also assessed the alpha diversity of the samples to explore potential differences in the microbial communities between the healthy eye and the anophthalmic socket. Alpha diversity, which measures the number of taxa and their abundance, was quantified using two key metrics: richness and the Shannon index. The Shannon index accounts for both the number of genera and the evenness of their abundances, offering insights into the overall diversity and complexity of the microbial community. Richness, in contrast, quantifies the total number of distinct genera present in a sample.

**Figure 2:**
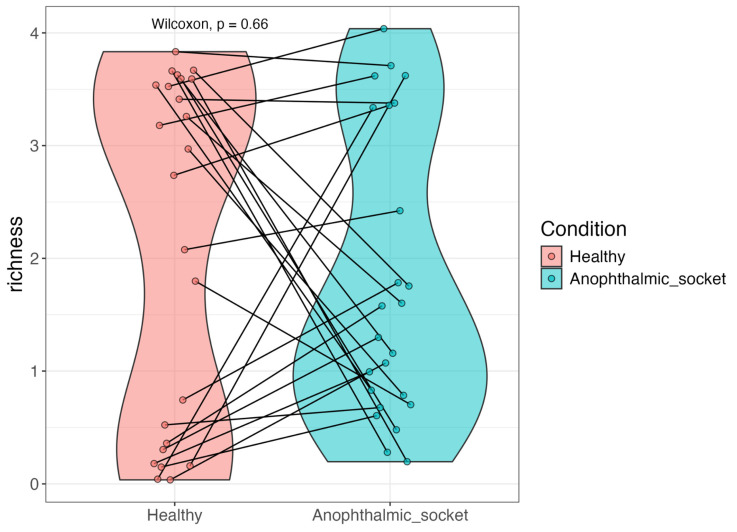
A violin plot illustrating the Shannon diversity index of the eye microbiota, comparing healthy eyes and their contralateral anophthalmic sockets. No statistical difference was found between the two groups of samples (*p*-value = 0.66).

The Shannon diversity index was employed to compare microbial diversity between healthy eyes and their contralateral anophthalmic sockets, as shown in Figure 2 with violin plots. A Wilcoxon signed-rank test, pairing each healthy eye with its corresponding anophthalmic counterpart, revealed no significant variations in terms of Shannon diversity. Both types of samples exhibited comparable levels of diversity, with no distinct trend indicating reduced diversity either within individual patients or across the cohort.

**Figure 3:**
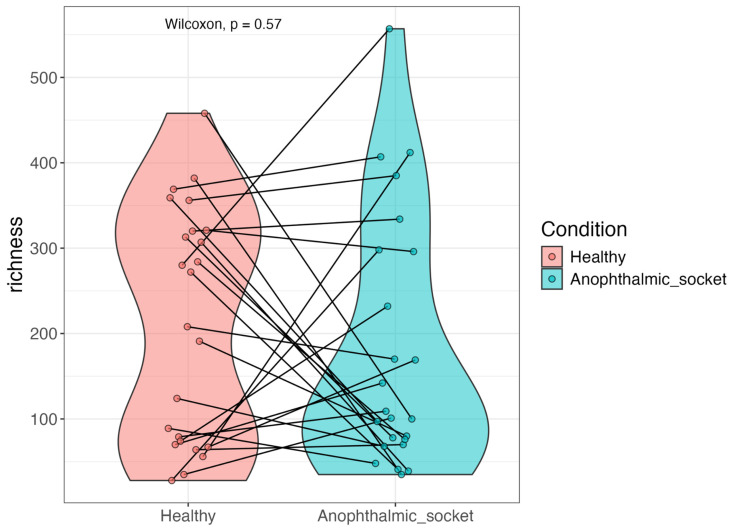
A violin plot illustrating the richness diversity index of the eye microbiota, comparing healthy eyes and their contralateral anophthalmic sockets. Left figure: T-test. Right figure: Wilcox test, paired. No statistical difference was found between the two (*p*-value = 0.57).

Subsequently, we calculated richness for each sample, following the same analytical procedure. Figure 3 also presents violin plots illustrating the richness index. Neither a T-test nor a Wilcoxon test revealed any significant differences between the two groups.

### 3.3. Beta Diversity Analysis

To understand the differences in microbial communities between distinct environments, we calculated the beta diversity. While alpha diversity focuses on within-sample diversity, beta diversity compares the composition between samples, highlighting how microbial communities vary across conditions.

**Figure 4:**
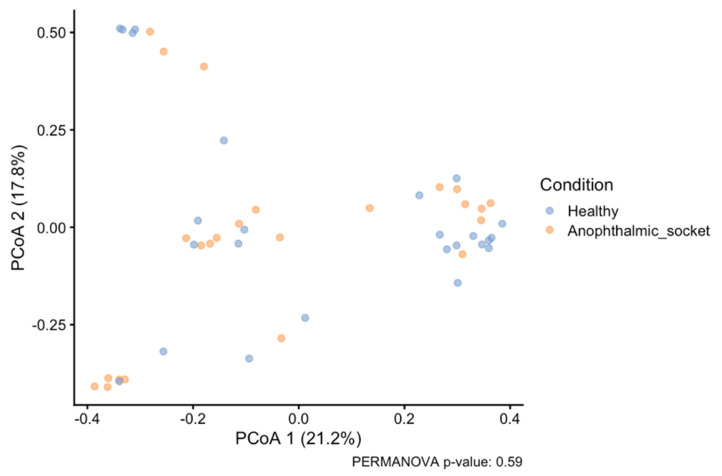
PCoA of samples using Bray–Curtis dissimilarity index. Two experimental groups are taken into consideration, healthy and anophthalmic socket samples (PERMANOVA *p*-value = 0.59).

The beta diversity Bray–Curtis dissimilarity index was used to compute the distance matrix between all samples, and the resulting data were visualized using principal coordinate analysis (PCoA) (Figure 4). The PCoA plot shows significant overlap between samples from healthy eyes and anophthalmic sockets, indicating that their microbial communities are not distinctly different. This observation is further supported by a PERMANOVA (*p*-value = 0.59), which suggests no statistically significant differences in microbial composition between the healthy eye and the anophthalmic sockets. These findings imply that, at the community level, the microbial populations in healthy eyes and anophthalmic sockets do not follow a specific and distinct taxonomic pattern.

### 3.4. Similarity to the Healthy Eye Population

**Figure 5:**
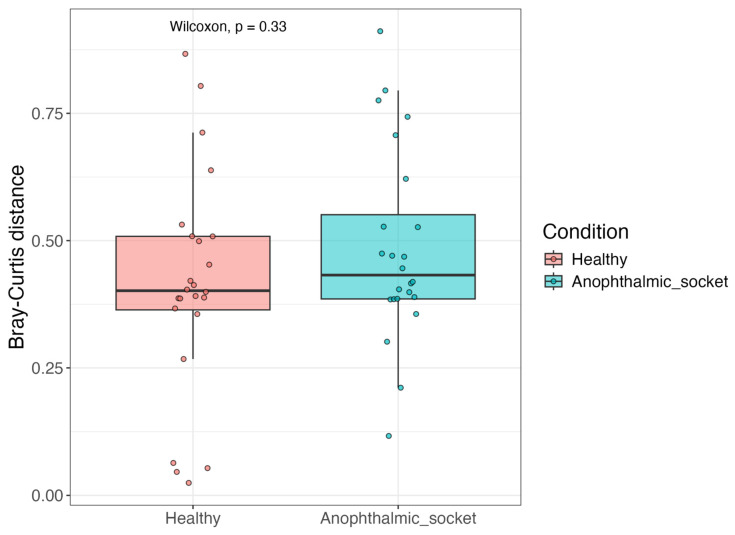
A boxplot representing the lowest Bray–Curtis Distance (BCD) comparing each sample to the healthy reference population of Borroni et al., 2022 [5].

To quantitatively assess the similarity of microbial communities in the studied samples compared to healthy eyes, we calculated the Bray–Curtis Distance (BCD) for each sample relative to a reference healthy eye population made of the samples available in Borroni et al., 2022 [5]. Figure 5 displays the minimum BCD value for each sample. Both healthy and anophthalmic socket samples exhibited a median dissimilarity of approximately 40% relative to the reference. Surprisingly, the similarity of anophthalmic sockets to the reference was comparable to that observed among healthy samples. This indicates that the microbial profiles of anophthalmic sockets are as similar or dissimilar to the reference as the profiles of other healthy samples, challenging the expectation that anophthalmic sockets would differ more significantly from healthy eyes.

### 3.5. Differential Abundance Analysis (DAA)

To identify significant differences in genus abundances between healthy eyes and anophthalmic sockets, we conducted a differential abundance analysis using DESeq2. This analysis aimed to pinpoint genera that were either over- or under-abundant in anophthalmic sockets compared to healthy eyes.

**Figure 6:**
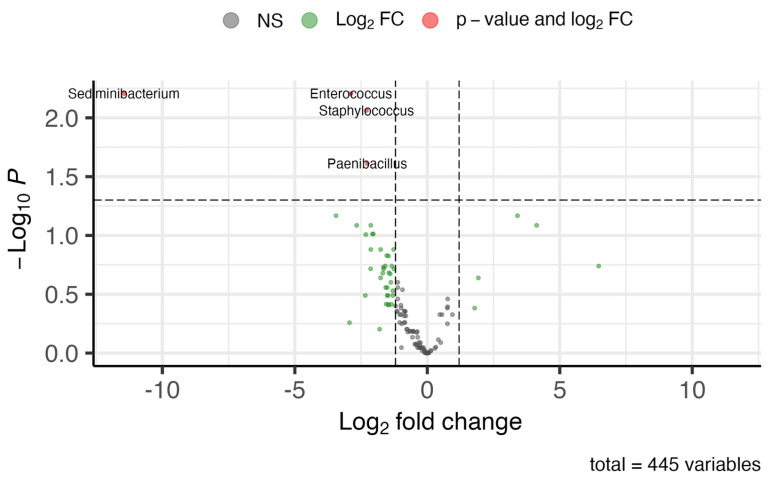
A volcano plot summarizing the differential abundance analysis (DAA) results with DESeq2. The following 4 genera appear to be under-abundant (less abundance in anophthalmic socket samples relative to healthy samples): Sediminibacterium, Enterococcus, Staphylococcus, and Paenibacillus.

**Figure 7:**
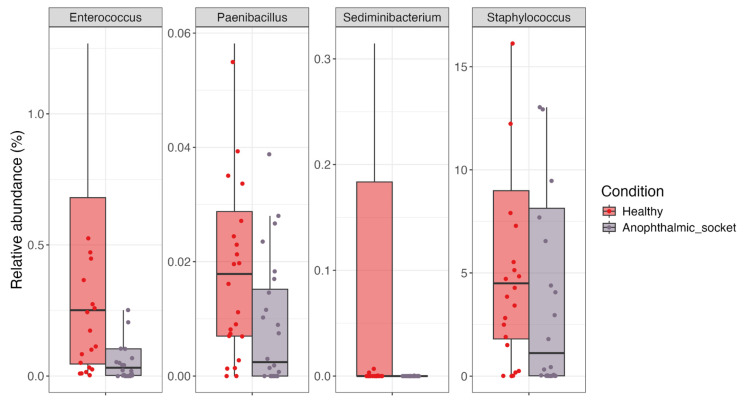
A boxplot representing the abundance of the 4 significant differential genera found by means of a DESeq2 analysis across sample groups.

The results, depicted in a volcano plot (Figure 6), highlighted four genera—*Sediminibacterium*, *Enterococcus*, *Staphylococcus*, and *Paenibacillus*—as under-abundant in anophthalmic socket samples. The analysis applied thresholds of an adjusted *p*-value below 0.05 and a log2 fold change below −1.2 or above 1.2. For a more detailed view, a boxplot (Figure 7) illustrates the relative abundance of these four differential taxa across all samples.

## 4. Discussion

The study of anophthalmic socket changes and its symptoms is gaining more and more importance nowadays. Anophthalmic socket syndrome is a well-known syndrome that may cause discomfort, discharge, and dryness, as well as other alterations in periorbital tissues. This syndrome has been proposed to be caused by multiple reasons such as reduced tear production, meibomian gland dysfunction (MGD) caused by lid margin abnormalities, loss of goblet cells, eyelid laxity, and lagophthalmos [13,14,15,16,17].

Other studies previously reported and suggested that there is a high frequency of dry anophthalmic socket syndrome (DASS) and a higher frequency of conjunctival inflammation in anophthalmic sockets compared to the fellow eye. These findings have been related to frequent prosthesis replacement (within a year), in addition to removing the prosthesis to clean it within a month. The continuous rubbing of the prosthesis against the conjunctiva could induce this inflammation. Besides this, tear deficiency could lead to hyperkeratinization and a reduced density of meibomian gland acinar units [18,19,20,21].

The OSM performs an important role in the defense mechanisms of the ocular surface system. It can modify immunological activity, constituting a barrier against pathogen invasion [7].

With the use of traditional techniques such as cultures, it has been demonstrated that the most common bacteria on the ocular surface are *Staphylococcus*, *Propionibacterium*, and *Corynebacterium*. At the phylum level, *Proteobacteria*, *Firmicutes*, and *Actinobacteria* have been proposed as dominant phyla in the ocular surface for several studies [22]. The genus *Pseudomonas*, a pathogen that causes ocular infections, has been identified in several studies as part of the OSM, suggesting that the ocular surface is normally exposed to the presence of potentially pathogenic microbes as well as fungi like *Aspergillus* [22,23]. Metagenomic techniques have allowed for the detection of a broader population, although the OSM remains a low-biomass niche, with a relatively stable, minimal core microbiome in which all individuals share a few taxa [6,22,24]. In fact, these techniques are also acquiring importance at the diagnostic level in clinical practice [25], such as in the diagnosis of bacterial keratitis, where shotgun sequencing is able to interrogate all microbial genomes in the eye microbiome in keratitis samples [11]. The use of these techniques allows for high levels of sensitivity and specificity and offers an added benefit in their ability to detect toxin-producing genes or other important biomarkers [26]. Surely, clinical correlations and assessments of treatment response remain imperative [27]. Our study showed that while the microbial community in an anophthalmic socket differs from that of its corresponding contralateral healthy eye, overall comparisons between healthy and anophthalmic socket samples did not reveal distinct clustering patterns in principal coordinate analysis (PCoA) or significant differences in a PERMANOVA. Alpha diversity metrics also did not show significant differences between the two groups. However, at a more detailed level, we observed specific changes in the abundance of genera such as *Staphylococcus*, *Enterococcus*, *Paenibacillus*, and *Sediminibacterium*. The general microbial profiles of the two eyes in the same patient differ, leaving it unclear whether these changes are a consequence of the pathology. Further studies are needed to analyze the microbiome profiles of both eyes in healthy individuals to determine whether such variability is present in the absence of any condition.

Several studies have explored changes in the ocular surface microbiome (OSM) in the presence of various pathologies and conditions. In the study by Borroni et al., samples from healthy eyes were categorized into nine ECSTs, with the vast majority of samples being highly enriched with *Staphylococcus*. Only a few samples were not dominated by *Staphylococcus*, with other genera such as *Bacillus*, *Pseudomonas*, and *Corynebacterium* predominating [5]. In contrast, most healthy eyes in our study were not classified into ECSTs 3, 4, or 5, which are characterized by a high enrichment of *Staphylococcus*. However, *Staphylococcus* was found to be significantly more abundant in the healthy eyes compared to the anophthalmic sockets.

In Rocha-de-Lossada et al., the authors found significant differences in the relative abundance of several phyla and genera in naïve keratoconus (KC), with the presence of the genera *Ralstonia* and *Pelomonas*, which were notably absent in the control group. They also noticed lower microbial richness and diversity in the KC group [2].

In Andersson et al., the authors compared the microbiota of healthy eyes against patients with aqueous tear-deficient dry eye (ADDE), and their findings indicated that the OSM is less diverse and aberrant in patients with ADDE, including having decreased relative abundances of several genera compared with controls [9]. In patients with dry eye disease, another study showed a different microbiome in comparison with the healthy eye, when the eyes remain closed [28]. In contrast, another study by Andersson J et al. could not prove that there were significant differences in bacterial diversity or composition for contact lens users versus non-contact-lens users [8].

The microbiome is considered crucial in maintaining the homeostasis of the ocular surface [29]. The study of the microbiome in dysbiotic states led to important discoveries in the pathophysiology and mechanisms of disease. Understanding the microbiome profiles associated with disease states can further our understanding of the variations in disease phenotypes and responses to treatment [24,30]. In addition, the existence of the gut microbiome is well-established knowledge, and associations between gut dysbiosis and eye diseases have been shown [31,32]. In fact, considering that the eye is a site of inflammatory diseases, it is possible that autoimmune reactions are associated with dysbiosis in the gut [12,33,34]. It has been proposed that environmental factors, particularly microbes, are vital in the development and progression of autoimmune diseases [35], although the direct interconnections between gut microbiota and eye function require a solid molecular foundation [36].

Recent studies have suggested that viral infections may also significantly impact the ocular surface microbiome. For example, SARS-CoV-2 has been detected on the ocular surface, raising the possibility that the eye could serve as a route of viral transmission. Additionally, viral infections may disrupt the ocular microbiome, exacerbating local pathology or contributing to systemic infections. Studies have shown that viruses, such as herpes simplex virus and influenza virus, can affect the ocular surface, and their presence may lead to imbalances in the microbial community, similar to what is observed with bacteria. These findings suggest that viral pathogens play a crucial role in both the pathogenesis of ocular surface diseases and the broader systemic implications of infection. Future research should explore the interactions between viral and bacterial microbiomes on the ocular surface to better understand the role of the microbiome in disease progression [37,38,39,40,41].

Research is ongoing in this area, and further studies are necessary to fully understand the specific micro-environmental impacts of microbiota diversity in AS patients and how these changes might contribute to DASS development and progression or offer therapeutic targets.

This study, while offering valuable insights into the ocular surface microbiota in anophthalmic socket patients, has several limitations that should be acknowledged. Firstly, the cross-sectional nature of this study means that it only provides a snapshot of the microbiota at a specific point in time, limiting the ability to assess dynamic changes over time. Longitudinal studies would be necessary to better understand the evolution of the microbiome in anophthalmic sockets and its potential role in the development of conditions such as dry anophthalmic socket syndrome (DASS). Additionally, the relatively small sample size may limit the generalizability of the findings, as microbiota composition can be influenced by a variety of factors such as geographical location, diet, and lifestyle. Another limitation is the lack of adjustment for potential confounders, such as age, gender, environmental exposure, or underlying systemic health conditions, all of which may influence the microbiota composition. Lastly, while this study focused on bacterial communities, it did not explore other components of the ocular surface microbiome, such as viruses and fungi, which may also play a significant role in ocular surface health and disease. Future research should aim to address these limitations by incorporating larger, more diverse cohorts, conducting longitudinal analyses, and broadening the scope to include viral and fungal components of the microbiome. Another limitation is that we conducted our analysis at the genus level due to the use of 16S rRNA sequencing, which does not allow for species-level resolution. For more accurate species identification, future studies could employ whole-genome sequencing (WGS) or full-length 16S rRNA sequencing. Future longitudinal studies with larger cohorts and more comprehensive controls are needed to validate our findings and explore potential mechanisms underlying the observed microbial differences.

In conclusion, the ocular surface microbiome (OSM) of anophthalmic sockets differs significantly from that of fellow healthy eyes. Our study identified several genera that are notably reduced in most anophthalmic sockets. Specifically, *Staphylococcus*, *Enterococcus*, *Paenibacillus*, and *Sediminibacterium* are less abundant in anophthalmic sockets relative to the healthy eye. This reduction could potentially influence the pathophysiology of DASS. Further research is needed to confirm these findings and elucidate their implications.

## Data Availability

The original contributions presented in the study are included in the article, further inquiries can be directed to the corresponding author.
